# Whole genome sequencing of *Mycobacterium tuberculosis* isolates and clinical outcomes of patients treated for multidrug-resistant tuberculosis in Tanzania

**DOI:** 10.1186/s12864-020-6577-1

**Published:** 2020-02-21

**Authors:** Bugwesa Z. Katale, Peter M. Mbelele, Nsiande A. Lema, Susana Campino, Stephen E. Mshana, Mark M. Rweyemamu, Jody E. Phelan, Julius D. Keyyu, Mtebe Majigo, Erasto V. Mbugi, Hazel M. Dockrell, Taane G. Clark, Mecky I. Matee, Stellah Mpagama

**Affiliations:** 1 Department of Microbiology and Immunology, School of Medicine, Muhimbili University of Health and Allied Sciences (MUHAS), Dar es Salaam, Tanzania; 2 Tanzania Wildlife Research Institute (TAWIRI), Arusha, Tanzania; 3 Kibong’oto Infectious Disease Hospital (KIDH), Sanya Juu, Tanzania; 4 Department of Global Health and Biomedical Sciences, School of Life Sciences and Bioengineering, Nelson Mandela African Institution of Science and Technology (NM-AIST), Arusha, Tanzania; 5 Field Epidemiology and Laboratory Training Programme, Dar es Salaam, Tanzania; 6 Faculty of Infectious and Tropical Diseases, London School of Hygiene &Tropical Medicine (LSHTM), Keppel Street, London, WC1E 7HT UK; 7 Department of Medical Microbiology, Catholic University of Health and Allied Sciences, Mwanza, Tanzania; 8 Southern African Centre for Infectious Diseases Surveillance (SACIDS), Sokoine University of Agriculture (SUA), Morogoro, Tanzania; 9 Department of Biochemistry, Muhimbili University of Health and Allied Sciences (MUHAS), Dar es Salaam, Tanzania; 10 Faculty of Epidemiology and Population Health, London School of Hygiene &Tropical Medicine (LSHTM), Keppel Street, London, WC1E 7HT UK

**Keywords:** Whole genome sequence, *Mycobacterium tuberculosis*, Multidrug-resistant tuberculosis, Time-to-culture-conversion, Treatment-outcomes

## Abstract

**Background:**

Tuberculosis (TB), particularly multi- and or extensive drug resistant TB, is still a global medical emergency. Whole genome sequencing (WGS) is a current alternative to the WHO-approved probe-based methods for TB diagnosis and detection of drug resistance, genetic diversity and transmission dynamics of *Mycobacterium tuberculosis* complex (MTBC). This study compared WGS and clinical data in participants with TB.

**Results:**

This cohort study performed WGS on 87 from MTBC DNA isolates, 57 (66%) and 30 (34%) patients with drug resistant and susceptible TB, respectively. Drug resistance was determined by Xpert® MTB/RIF assay and phenotypic culture-based drug-susceptibility-testing (DST). WGS and bioinformatics data that predict phenotypic resistance to anti-TB drugs were compared with participant’s clinical outcomes. They were 47 female participants (54%) and the median age was 35 years (IQR): 29–44). Twenty (23%) and 26 (30%) of participants had TB/HIV co-infection BMI < 18 kg/m^2^ respectively. MDR-TB participants had MTBC with multiple mutant genes, compared to those with mono or polyresistant TB, and the majority belonged to lineage 3 Central Asian Strain (CAS). Also, MDR-TB was associated with delayed culture-conversion (median: IQR (83: 60–180 vs. 51:30–66) days). WGS had high concordance with both culture-based DST and Xpert® MTB/RIF assay in detecting drug resistance (kappa = 1.00).

**Conclusion:**

This study offers comparison of mutations detected by Xpert and WGS with phenotypic DST of *M. tuberculosis* isolates in Tanzania. The high concordance between the different methods and further insights provided by WGS such as PZA-DST, which is not routinely performed in most resource-limited-settings, provides an avenue for inclusion of WGS into diagnostic matrix of TB including drug-resistant TB.

## Background

Tuberculosis (TB), caused by the *Mycobacterium tuberculosis* complex (MTBC), is the leading cause of mortality, killing about 2 million people each year globally [1]. The highest TB mortality and morbidity occurs in low and middle income countries like Tanzania [1, 2]. HIV infection is the most important single predictor of TB incidence across the African continent [3]. This is critical because HIV/AIDS is likely to increase in the risk of progression TB infection by 30 times, due to impairment on the immune system [3]. Sub-Saharan Africa carries a disproportionate burden of HIV, accounting for more than 70% of the global burden of infection [4]. For instance, in 2017, nearly 2.5 million people who contracted TB lived in sub-Saharan Africa, and 665,000 of them died from the disease [5]. The recent introduction of the Xpert®MTB/Rif assay (Cepheid, USA) has shown an increase in detection of drug resistance TB patients and the detection of multidrug-resistant TB (MDRTB) has increased three- to eight-fold compared to conventional testing [6]. Despite the gaps in documentation of MDR-TB cases in several Sub-Saharan African countries (SSA), pooled analysis that involved several studies reported a prevalence of 2.1% of MDR-TB in new cases, signifying a low prevalence of MDR-TB cases in SSA [7]. The relatively low prevalence of MDRTB in SSA might be attributed by the recent introduction of rifampicin in Africa, by the use of rifampicin-free treatment regimens in the continuation phase (during months three to eight), by the growing use of directly observed treatment as recommended under the directly observed treatment, short course (DOTS) strategy, and by the use of fixed-dose combination tablets in a few countries [8, 9].

Tanzania is among African countries south of Sahara with the highest burdens of TB, with an estimated 295 TB cases per 100,000 adults. The National anti-tuberculosis drug resistance survey conducted in 2010 in Tanzania found the resistance to any of the four first-line drugs in new patients to be 8.3%, while the prevalence of MDR-TB was 1.1%. However, the crude prevalence for any resistance and for MDR-TB in retreated cases increased to 20.6 and 3.9% respectively [10].

The emergence of resistant strains of MTBC to anti-tuberculosis drugs like rifampicin (RIF), isoniazid (INH), pyrazinamide (PZA), ethambutol (EMB), streptomycin (SM) among others; and the emergence of HIV in the 1980s led to resurgence of TB [11]. Multidrug resistant TB (MDR-TB), defined as resistance to at least RIF and INH, is usually caused by MTBC strains that harbour mutations in *rpoB*, *katG* and *inhA* genes associated with RIF and INH resistance. Globally, MDR-TB claims over 580,000people’s lives annually [12]. About 10% of MDR-TB isolates exhibit resistance to fluoroquinolones (FQs) and second-line-injectable drugs (SLIDs) (aminoglycosides/cyclic-polypeptides), referred to as extensive drug resistant TB (XDR-TB), which challenges clinical management [2, 13, 14]. Patients with XDR-TB are infected with MTBC that have mutations in *rrs, tlyA or eis* promoter region (SLI related) and *gyrA* or *gyrB* genes (FQ related). Clinical management of both MDR-TB and XDR-TB is very complex and there are different treatment regimens [15]. About 50 and 70% of patients treated for MDR-TB and XDR-TB have unfavourable treatment outcomes, respectively. Although XDR-TB is rare in Tanzania, cohort review reports of patients treated for MDR-TB show that treatment success is only 75%. However, control and prevention measures should be strengthened in the country to reduce MDRTB related morbidity and mortality. To improve treatment outcomes and control escalation of MDR/XDR-TB; early, rapid and accurate diagnostic methods are required to detect and decipher susceptibility profile of the MTBC to anti-TB drugs in TB endemic settings such as Tanzania.

As in other TB endemic settings, Tanzania has deployed rapid molecular methods for dual detection of MTBC and susceptibility to either RIF alone or along with INH and second-line injectable and fluoroquinolones. For example, while the Xpert®MTB/Rif assay (Cepheid, USA) detects MTBC and provides information about susceptibility to RIF only [16], the genotype MTBDRplus and MTBDRsl assays (Hain Life sciences, Germany) detect mutations that are strongly associated with multiple types of MDR-TB and also XDR-TB respectively [17, 18]. These assays have potential to guide implementation of the new WHO shorter regimen for treating MDR-TB [15, 19]. Unlike Whole genome sequencing (WGS), these PCR-probe based assays have limited anti-TB drug-susceptibility value especially if mutations occur outside the target region [20]. In recent years, WGS platforms have become an alternative diagnostic in addressing probe-based assay limitations. WGS can identify genotypes predictive of drug-resistance phenotype within the entire region of microbial genome and has potential to determine genetic relatedness and identify transmission dynamics necessary in guiding clinical decisions. There is a need to sequence as many MTBC strains as possible, build libraries of single nucleotide polymorphisms (SNPs) and other variants, compare the relatedness of MTBC strains, and correlate variation with clinical progress or outcomes as a whole. Based on this, we performed WGS for the first time on isolates sourced from patients treated for MDR-TB at Kibong’oto Infectious Diseases Hospital (KIDH) in Tanzania. These data will provide a baseline set of the types and variations in TB in Tanzania. Clinical and laboratory information such as treatment regimen, phenotypic susceptibility profile, prior history of treatment with first line anti-TB drugs, HIV status, culture conversion rate and treatment outcomes were available for analysis.

## Results

### Baseline demographic and clinical characteristics of study participants

A total of87study participants with positive MTBC culture results were included in the genetic study of whom, 47 (54%) were females. The median age was 35 (Interquartile range = 29–44) years (Table 1). Twenty (23%) were co-infected with HIV and had a median CD4 counts of 246 (IQR =119–388) cells/mm^3^.

**Table 1:** Demographic and clinical information of study participants

Variable	N	%
Gender		
Male	40	46
Female	47	54
Age category (years)		
< 33	31	35.6
≥ 33	56	64.4
HIV status		
Negative	67	77
Positive	20	23
Diabetic		
Yes	1	1.2
No	86	98.8
Previous history of TB treatment		
New	45	51.7
Retreatment	42	48.3
CD4 count (*n* = 20)		
< 200	8	40
≥200	12	60
Body mass index		
< 16–18.5	26	29.9
≥ 18.5	61	70.1

#### Association of clinical information with genetic drug resistance

Table 2 summarizes demographic and clinical characteristics of TB patients. We found association between previous history of TB treatment and phenotypic drug resistance (OR, 0.01 95% CI: 0.0003–0.0995, *p* = < 0.001) (Table 2).

**Table 2:** Demographic and clinical characteristics of drug resistance TB patients

Variables, n (%)	DR-TB (*n* = 57)	DS-TB (*n* = 30)	OR (95% C.I) for DR-TB	*p* values
Gender				
Male	26	14	o.96 (0.36–2.56)	0.93
Female	31	16		
Age (years)				
< 33	21	10		
≥ 33	36	20	1.17 (0.42–3.34)	0.55
HIV status				
Negative	41	26	2.5 (0.70–11.47)	0.12
Positive	16	4		
CD4 count (n = 20)				
< 200	6	2		
≥ 200	10	2	0.6 (0.04–10.62)	0.65
Previous history of TB treatment				
New	16	29	0.01 (0.0003–0.0995)	<0.001
Retreatment	41	1		
Body mass index				
< 16–18.5	19	7		
≥ 18.5	38	23	1.6 (0.55–5.34)	0.33

### Analysis of mutations associated with phenotypic drug resistance to anti-TB drugs

﻿Of the 87 MTBC isolates sequenced, 57 (65.5%) had at least one mutation in a gene that was predictive for 7 anti-drugs (Table 3). The concordance between phenotypic DST and WGS was 97% for RIF (DST: WGS; 26:28 isolates), 81% INH (DST: WGS; 40:51 isolates) and 95% for SM (DST: WGS; 8:11 isolates) (kappa = 1.00). Moreover, the concordance between Xpert® MTB/RIF (Cepheid, USA) and WGS for detection of RIF resistance was 95% (8:11 isolates) (kappa = 1.00). Substitution of serine to threonine at codon 315 (S315T) of the *katG* gene accounted for 50 (94.3%) of mutations (Table 3). The common mutation associated with phenotypic resistant to RIF was S450L (substitution of serine to leucine) accounted for 96% (26/27) of the detected RIF resistance. Of the 57 isolates, 29 (33%) had mutations in the *embB* gene and Q497R was the common mutation (8/29; 27.6%) (Table 3). Nineteen isolates had mutations, in *pncA* genes of which V128G was the common accounting for 7 (36.8%) (Table 3). In addition, our analysis revealed pncA deletion involving Rv2044c in two drug resistance isolates (MDRTB & polyresistance isolates) that contributed 5.3% (1 isolates) of MDRTB and other resistance pattern (Table 3). Patients with MDR-TB had highly diverse mutations as compared to monoresistance (Table 3). We found 2 pre patients harbouring XDRTB isolates. The drug resistance mutations involving either rrs or gyrB genes against AMK and FQs respectively were detected in low proportions among DR resistance isolates (Table 3). These isolates had mutation either at the *rrs* gene involving substitution of C to T at codon 51 (C517T) or *gyrB* gene which involved replacement of R by C at codon 446 (R446C). MTBC isolates from drug susceptible patients had no mutations in genes encoding for INH, RFP, PZE, STM and EMB.

**Table 3:** Frequency and distribution of mutations associated with drug resistance *M. tuberculosis*

Drug	Target gene	mutation	n (%) in monoresistance	n (%) in MDR-TB strains	n (%) in other resistance pattern	Total (N)
RIF (*n* = 27)	*rpoB*	S450L	4 (14.8)	16 (59.3)	6 (22.2)	26
		D435Y	1 (3.7)	1 (3.7)	–	1
INH (*n* = 55)	*katG*	S315T	10 (18.2)	24 (43.6)	16 (29.1)	50
		S302R	–	1 (1.8)	1 (1.8)	2
		N138S	–	1 (1.8)	–	1
	*oxyR’-ahpC*	C52T	–	1 (1.8)	–	1
		C54T	–	1 (1.8)	–	1
EMB (*n* = 29)	*embB*	Y319C	–	–	1 (3.4)	1
		Q497R	–	6 (20.9)	2 (6.9)	8
		G406D	–	–	1 (3.4)	1
		G406S	–	2 (6.9)	3 (10.3)	5
		M306L	–	1 (3.4)	–	1
		M306I	–	1 (3.4)	–	1
		G406A	–	3 (10.3)	–	3
		M306V	–	2 (6.9)	–	2
		Y334H	–	1 (3.4)	–	1
		M306T	–	1 (3.4)	–	1
	*EmbC-embA*	C16T	1	1 (3.4)	3 (10.3)	5
PZA (*n* = 19)	*pncA*	V128G	–	8 (42.1)	–	8
		A193AT	–	–	1 (5.3)	1
		G17D	–	–	1 (5.3)	1
		D8N	1	–	–	1
		L85P	–	–	1 (5.3)	1
		A46V	–	1 (5.3)	–	1
		P69L	–	1 (5.3)	–	1
		Q10R	–	1 (5.3)	–	1
		D49G	–	1 (5.3)	–	1
		D63A	–	1 (5.3)	–	1
		Rv2044c	–	1 (5.3)	1 (5.3)	2
STR (n = 3)	*rpsL*	K43R	–	2 (100)	–	2
	*rrs*	C517T	–	1 (100)	–	1
FLO (n = 1)	*gyrB*	R446C	–	1 (100)	–	1
AMK (n = 2)	*rrs*	C517T	–	2 (100)	–	2

#### Treatment outcome and culture conversion rates among the drug resistance TB isolates

Of the 57 study participants with mutation (s) to at least one of the anti-TB drugs, 47 (82.5%) had successful treatment, 3 (5.3%) died, and 7 (12.3%) defaulted (Table 4). Three patients (2MDR-TB and 1 with polyresistant) died 9 months after enrolment, during which they were in a continuous phase of anti-mycobacterial medications. The median (IQR) culture conversion rates were 51 (30–66) days and 83 (60–180) days for participants with mono/polyresistant and MDR/XDR-TB, respectively (Table 4).

**Table 4:** Drug resistance TB patients’ susceptibility testing, genotypic resistance, culture conversion rate and treatment outcome (*n* = 57)

Drug pattern resistance (*N* = 57)	Type of mutation in drug target genes										Phenotypic DST	Mean culture conversion in days	Treatment outcome		
	**INH**		**RIF**	**EMB**		**PZA**	**SM**		**FQ**	**AMK**			**Success**	**Defaulted**	**Died**
	***katG***	***oxyR’-ahpC***	***rpoB***	*embB*	***embC-embA***	***pncA***	***rpsL***	***rrs***	***gyrB***	***rrs***					
Monoresistance (*n* = 17)	S315T	C54T	S450L	M306I	C16T	D8N	K43R	C517T			resistance	51	16	1	0
MDR-TB (*n* = 24)	S315T N138S		S450L D435Y	Q497R M306I M306T G406A M306V M306L G406S Y334H	C16T	P69L Rv2044c V128G	K43R	C517T	R446C	C517T	resistance	83	17	5	2
Others e.g. polyresistance (*n* = 16)	S315T S302R	C52T		Q497R G406D G406S M306I	Y319C C16T	G17D L85P Rv2044c D49G D63A	K43R			C517T	resistance	66	14	1	1

### Phylogenetic analysis of the drug resistance TB isolates

**Fig. 1:**
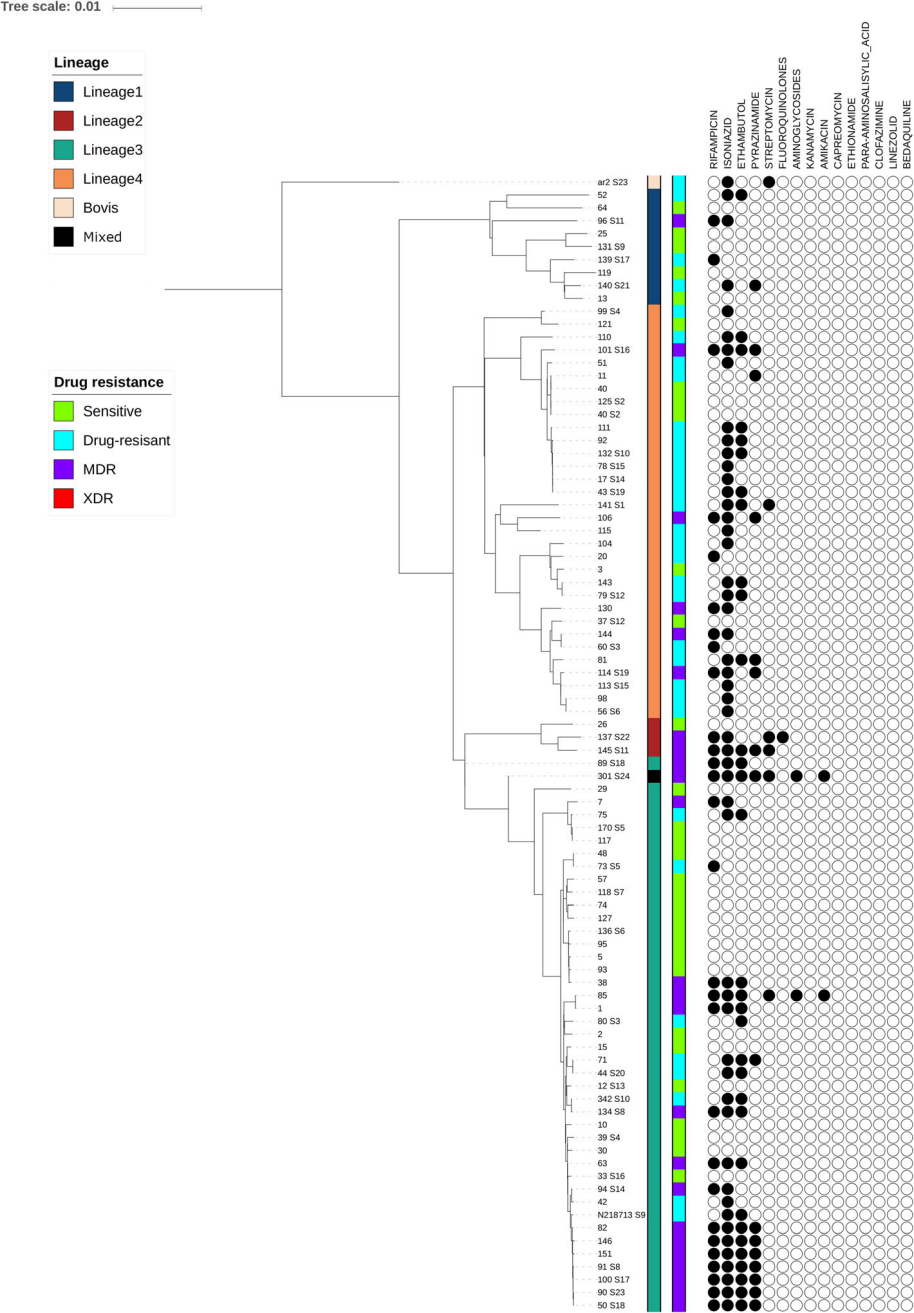
Phylogenetic tree showing relationship of *M. tuberculosis* drug resistance strains, and pattern of drug resistance

Phylogenetic analysis revealed that the DR-TB strains were heterogeneously distributed in lineages 1 to 7 (Fig. 1). The Central Asian Strains (CAS) (lineage 3) predominated in the MTBC strains and accounted for 48.3% (42/87) of the isolates, followed by lineage 4 (32/87; 36.8%), lineage 1 (10/87; 11.5%), lineage 2 (3/87; 3.4%) and 1 (1.1%). Of the 24 MDR isolates, 15 (62.5%) isolates belonged to lineage 3 (CAS), 5 (20.8%) isolates were lineage 4 (Latin American Mediterranean, LAM), 2 (8.3%) isolates were lineage 2 and 2 (8.3%) isolates originated from lineage 1 (East African Indian, EAI) (Table 5).

**Table 5:** Description of lineages, spoligotype, cluster of drug resistance *M. tuberculosis* and genotype at important drug loci

Isolate ID	Lineages	Spoligotype	WGS cluster	Genotype at drug loci									
				RIF	ZN	AMK	PZ	FLR	AMG	EMB		ST	
				*rpoB*	*katG*	*rrs*	*pncA*	*gyrB*	*rrs*	*embB*	*embC-embA*	*rpsL_*	*rrs*
1	lineage3	CAS	MDR	S450L	S315T		Large deletion pncA Rv2044c						
7	lineage3	CAS	MDR	S450L	S315T								
11	lineage4	LAM	DR				D8N						
20	lineage4	LAM	DR	D435Y									
38	lineage3	CAS	MDR	S450L	N138S					M306V, Q497R, G406A			
42	lineage3	CAS	DR		S315T								
51	lineage4	LAM	DR		S315T								
52	lineage1	EAI	DR		S315T					Y319C			
63	lineage3	CAS	MDR	S450L	S315T					M306I			
71	lineage3	CAS	DR		S315T		G17D			Q497R			
75	lineage3	CAS	DR		S315T					G406D			
81	lineage4	LAM	DR		S315T		L85P			G406S, M306I			
82	lineage3	CAS	MDR	S450L	S315T		V128G			M306L			
85	lineage3	CAS	MDR	S450L	S315T	C517T			C517T	G406A			C517T
92	lineage4	LAM	DR		S315T					Q497R, G406S			
98	lineage4	LAM	DR		S315T								
104	lineage4	LAM	DR		S315T								
106	lineage4	LAM	MDR	S450L	S315T		Q10R						
110	lineage4	LAM	DR		S315T								
111	lineage4	LAM	DR		S315T								
115	lineage4	LAM	DR		S315T, S302R								
130	lineage 4	LAM	MDR	S450L	S315T								
143	lineage 4	LAM	DR		S315T						C16T		
144	lineage 4	CAS	MDR	S450L	S315T								
146	lineage 3	CAS	MDR	S450L	S315T		V128G						
151	lineage 3	CAS	MDR	S450L	S315T		V128G			Q497R			
100-S17	lineage 3	CAS	MDR	S450L	S315T		V128G			Q497R			
101-S16	lineage 4	LAM	MDR	S450L	S315T, S302R		D49G			Q497R			
113-S15	lineage 4	LAM	DR		S315T								
114-S19	lineage 4	LAM	MDR	S450L	S315T		D63A						
132_S10	lineage 4	LAM	DR		S315T								
134_S8	lineage 3	CAS	MDR	S450L	S315T					G406S			
137-S22	lineage2	Beijing	MDR	S450L	S315T			R446C					
139-S17	lineage 1	EAI	DR										
140-S21	lineage1	EAI	DR		OxyR-ahpC, C52T, large deletion, S315T		A193AT						
141-S1	lineage 4	LAM	DR		S315T		Large, deletion, pncA-Rv2044c						
145-S11	lineage 2	Beijing	MDR	S450L	S315T		P69L			M306T, G406A		K43R	
17-S14	lineage 4	LAM	DR		S315T								
301-S24	lineage 3	CAS	MDR	S450L	S315T	C517T	A46V		C517T	M306G, G406S			
342-S10	lineage 3	CAS	DR		S315T					G406S			
43-S19	lineage4	LAM	DR		S315T								
44-S20	lineage 3	CAS	DR		S315T								
50-S18	lineage 3	CAS	MDR	S450L	S315T		V128G			Y334H,	C16T		
56-S6	lineage 4	LAM	DR		S315T								
60-S3	lineage 4	LAM	DR	S450L									
73-S5	lineage3	CAS	DR	S450L									
78-S15	lineage4	LAM	DR		S315T								
79-S12	lineage4	LAM	DR		S315T						C16T		
80-S3	lineage3	CAS	DR							M306I	C16T		
89-S18	lineage4	LAM	MDR	S450L	S315T								
90-S23	lineage 3	CAS	MDR	S450L	S315T		V128G			Q497R, M306V			
91-S8	lineage 3	CAS	MDR	S450L	S315T		V128G,			Q497R			
94-S14	lineage 3	CAS	MDR	S450L	S315T								
96-S11	lineage1	EAI	MDR	S450L	S315T								
99-S4	lineage 4	LAM	DR		S315T								
S23	lineage		DR		S315T						C16T	K43R	
S9	lineage 3	CAS	DR		S315T								

*RIF* Rifampicin, *INH* Isoniazid, *AMK* Amikacin, *PZ* Pyrazinamide, *FLR* Fluoroquinolones, *AMG* Aminoglycosides, *EMB* Ethambutol, *ST* Streptomycin

## Discussion

In this study, we found high concordance between WGS and conventional culture-based DST in predicting phenotypic drug resistance to anti-TB drugs, ranging from 81% for INH to 97% for RIF, and 95% concordance with Xpert® MTB/RIF (Cepheid, USA) for RIF. These findings are in agreements with those previously reported in different TB endemic settings [21–24]. The ability of Xpert® MTB/RIF and phenotypic detection of TB drug resistance cannot be underscored as it can pave a way to complementary and confirmatory WGS in early detection of resistance. Studies have shown phenotypic susceptibility tests to serve as reference standards [25], and that both Xpert assay and DST can provide information on polyresistance in pre- XDR-TB [26] despite varying sensitivities [27, 28].

The current practice in diagnosing drug resistant tuberculosis is through the WHO-approved probe-based assays like Xpert® MTB/RIF (Cepheid, USA) for RIF [16] and either genotype MTBDRplus for RIF and INH or genotype MTBDRsl (Hain Lifesciences, Germany) for aminoglycosides/capreomycin and fluoroquinolones that must be confirmed with culture-based DST [29]. Unlike WGS, these assays not only have limited DST range to identify hot-spot resistant determining regions but they also cannot inform transmission dynamics [30, 31]. The high concordance supports adoption of WGS as an alternative diagnostic tool to complement results from DST of all previously treated presumptive or confirmed rifampicin resistance cases in clinical settings. Although the facilities are not yet available the approach can be advocated in future clinical settings to enable reduction in unnecessary laboratory diagnostic time delays between identification of patients suspected of MDR-TB and initiation of treatment [29]. The potential for WGS to provide a rapid and comprehensive view of the genotype and reliable prediction of the drug susceptibility phenotype has been reported elsewhere [23, 32, 33]. This is an important platform to inform about susceptibility profile drugs like pyrazinamide (PZA), which is prescribed empirically in Tanzania and other resource limited countries. PZA kills semi-dormant bacilli and has synergetic activity with bedaquiline (BDQ), a key core drug in the currently recommended all-oral injectable free MDR-TB regimen(s). This synergism supports design of a shortened treatment regimens, which favours healthy patient outcomes [34].

In our study, the WGS uncovered 2 pre patient harbouring XDR-TB isolates, which were also detected by phenotypic DST at our hospital and later cured after MDR-TB treatment. This finding adds value to WGS as a potential molecular tool to complement phenotypic-DST and other molecular assay such Gene Xpert® MTB/RIF (Cepheid, USA) MDR-TB results for informed decision-making prior to anti-tuberculosis therapy. For effective use particularly in low- and middle-income countries, WGS must be robust, easy to use, and affordable.

We also found that the majority of the MTBC isolates from DR-TB participants had mutations in genes predictive of phenotypic resistance to all 7 anti-TB drugs tested including INH (*katG*), RIF (*rpoB*), EMB (*embB, embC-embA*), FQs (*gyrB*) and second line injectable drugs (*rrs*) (Table 3). The commonest mutations found in this study was S450L for *rpoB* (26/27), S315T on *katG* (51/56), Q497R (8/29) and G406S (5/29) on *embB*, C16T (5/29) on *embC-embA*, V128G (7/19) on *pncA* and K43R on *rpsL* genes are in keeping with findings reported elsewhere [35, 36]. As expected, patients with MDR-TB had multiple mutations in these genes, confirming the previous concept that drug resistance in mycobacteria spp., and other bacteria evolves as they accumulate mutations either de novo or after multiple and longer exposure to antibiotics [37, 38]. This high diversity of mutations among DR-TB patients suggests on-going transmission of MDR-TB strains rather than acquisition through random mutation and selection of drug resistance strains [37, 39]. In addition, our analysis revealed a large deletion that involved Rv2044c in the PZA resistance isolates. Such isolates also possessed mutations either in drug resistance genes for RIF or INH. INH, FQS and SLIDs are used for shorter MDR-TB treatment regimes. However, mutations involving genes associated with INH, EMB, FQs and SLIDs resistance affect the selection of appropriate treatment regimens [15, 19]. INH and EMB have been in use for several decades either as part of the standard treatment regimen for drug susceptible TB and INH monotherapy as infection preventive therapy for HIV infected individuals [40]. On the other hand, MTBC isolates from DS-TB participants had no mutations in genes encoding for INH, RIF, PZA, SM and EMB, which is in keeping with Cryptic consortium findings that reported susceptibility profiles for all isolates unless uncharacterized mutations or missing key nucleotide calls were present [41]. We found 2 pre-XDR-TB patients possessing R446C and C517Tmutations in the *gyrB* and *rrs* genes, in addition S315T, S450L, G406A and C517T mutations in the *katG*, *rpoB*, *embB* and *rrs* genes respectively. At this juncture, it is worthwhile to translate these mutations into clinical practice and investigate their association with participant’s health outcomes. In this study, participants with MDR-TB were cured using the recommended treatment regimen and reverted late to culture negativity as compared to those with mono- or polyresistance (Table 4). Time to culture conversion was highly prolonged to more than 6 months in participants with pre-XDR-TB, who had multiple mutations. Delayed time to culture conversion in our study is similar to findings by Sangita et al., [42] and Shibabaw et al., [43] in India and Ethiopia who documented reversion time to culture negativity after 125 and 77 days of treatment respectively. In addition, the spectrum of mutations to genes that predict phenotypic resistance to anti-TB drugs determines treatment outcomes. Fortunately, 82.5% (47/57) of study participants achieved treatment success, similar to findings by Meressa et al., [44] in Ethiopia. This success rate is 30% higher than the global MDR-TB treatment success rate [15], positioning the WGS method along with culture based DST as a useful testing algorithm for rigorous microbiological monitoring that will accelerate the 2035 strategic END-TB vision to create a world population free of TB [45]. Over 17% of study participants had unfavourable treatment outcomes (5% mortality and 12% lost-to- follow-up), similar to that reported by Meressa et al. [44],. However, this is lower than that reported by Dheda et al., [46] and Milanov et al., [47] in South Africa and Bulgaria who both documented over 50% unfavourable treatment outcomes. Previous findings have argued that severe malnutrition (BMI < 16 kg/m2), HIV co-infection, previous history of anti-TB therapy and drug adverse events can predict poor treatment outcomes [47, 48]. In this study, 29.9% of the study participants had low BMI and 23% were infected with HIV but had no effects on MDR-TB treatment outcomes.

In our study, the phylogenetic analysis showed distinct DR-TB spoligotype lineages similar to a previous finding by Kidenya et al. [49] in North-western Tanzania who reported strain variation across different spoligotype-defined *M. tuberculosis* lineages. We found that, the Central Asian strains (CAS) genotype (lineage 3) were the predominant lineages (Table 5), with no evidence of any change in terms of the CAS dominance over the past few years in Tanzania [50]. The CAS is among the prevalent lineage in the Indian subcontinent, South-East Asia, the Middle-East and East-Africa [51], showing a North-South divide along the Tropic of Cancer in the Eastern hemisphere – mainly in Asia, and partly prolonged along the horn of Africa [51, 52]. The dominance of CAS lineages in our local settings might be attributed to early contact due to migration and trade between people in Asian and East Africa countries. The seemingly prolonged stable dominance of CAS lineage reflects limited movement in of new infections from other sources/origins to influence the constantly circulating strains.

The main strength of our study is that we have correlated and linked WGS with treatment response and outcomes in a resource limited clinical TB endemic setting like Tanzania, which was not available previously. WGS provides high resolution when investigating diseases transmission in outbreaks, provides results for 2nd line drugs which is currently not performed in our settings. In addition to that, the WGS enables earlier use of the most appropriate drug regimen, thus improving patient outcomes and reducing overall healthcare costs [33]. This is the first step to start thinking how this technology could be included and add value in the future DR-TB diagnosis cascade, in high burden areas with limited resources where the need for rapid and accurate tools for assisting clinical decision making for optimal patient care and in predicting the treatment outcomes is very high.

However, our study has some limitations. Phenotypic culture-based DST for PZA, and cycloserine, which were part of the standardized treatment regimen for all participants was missing. In addition, concordance between line probe assays (LPA) genotype MDBTDRplus and MTBDRsl and WGS could not be done, which would have given a full comparison of the robustness of the WGS since the former had not been in use at the time of the study. In addition, WGS was performed retrospectively on clinical isolates, genetic data was not applied in the real time in the clinical setting, and hence no treatment regimen was modified to favour outcomes. Moreover, the use of WGS on frozen isolates of *M. tuberculosis* does not allow us to make considerations and comparisons between Xpert®MTB/Rif assay, phenotypic DST and WGS. Finally, WGS provides rapid and comprehensive diagnosis of DR-TB and the WHO has recognized its great potential for rapidly diagnosing drug-resistant TB in diverse clinical settings. However, there are challenges that need to be overcome prior to the implementation of whole-genome sequencing in resource-limited countries. These include high cost of equipment, the requirement for technical training, the need for expert guidance on the clinical interpretation of WGS data as the kind of information provided from the previous typing methods, the lack of simple solutions to obtain genome sequencing information directly from sputum samples and how it can be accommodated into our pre-existing diagnostic frameworks. Nevertheless, the useful information presented in this study outweighs these limitations and can be used to advocate for a policy change in the adoption of molecular TB diagnostic algorithms.

## Conclusion

WGS uncovered mutations in genes that predict phenotypic resistance to anti-TB drugs, signifying its importance in informed decision making prior to anti tuberculosis therapy. Expectedly, WGS was robust in ruling out DR-TB especially in absence of detectable mutations, which was the case in drug susceptible *M. tuberculosis* clinical isolates. The WGS-based DST best correlated with culture conversion rates and treatment outcome, indicating its potential application in designing an optimally individualized treatment regimen for favourable treatment outcomes in TB endemic settings.

## Methods

### Study settings

This study was conducted at Kibong’oto Infectious diseases hospital (KIDH) in Siha District, Kilimanjaro region, Tanzania. KIDH is the national centre of excellence for clinical management of drug resistant TB in the country and has a bed capacity of 320. The hospital provides TB services to more than 150 and 500 patients with drug resistant and susceptible TB per year, respectively. Recruitment and sputa collections from study participants were done at KIDH. Xpert® MTB/Rif assay, smear microscopy for acid-fast-bacilli (AFB) and isolation of MTBC on Lowenstein-Jensen (LJ) solid medium was performed at KIDH Mycobacteriology laboratory. MTBC isolates were transported to the Central Tuberculosis Reference Laboratory (CTRL) located at Muhimbili National Hospital (MNH) in Dar-es-salaam, Tanzania, for phenotypic susceptibility testing (DST) to first- and second-line anti-TB drugs like RIF, INH, EMB, SM, ofloxacin (OFX) and Kanamycin/Amikacin (KAN/AMK) and DNA extraction. Due to its complexity, PZA-DST is not routinely performed in Tanzania. MTBC DNA were shipped to London School of Hygiene and Tropical Medicine (LSHTM) for WGS and bioinformatics analysis.

### Study design, population and recruitment of participants

This study involved 57 and 30 participants who harboured *M. tuberculosis* that were resistant and susceptible to anti-TB drugs respectively, presenting at KIDH during the study period in 2014. Participants with resistant isolates were recruited if they had laboratory results by either GeneXpert MTB/RIF or phenotypic culture and DST [16]. The drug resistance was either mono/polyresistance-resistant or MDR-TB. The rest were recruited if they were infected with MTB that was susceptible to RIF by Xpert® MTB/Rif assay. Eligibility included age ≥18 *years*, and willingness to sign a written informed consent and provide sputum samples for laboratory analysis. Very sick participants were excluded from the study.

### Data collection

A standardized semi-structured questionnaire containing a set of study variables was used to collect data from study participants and medical charts were used to monitor treatment. Pre-treatment data that was collected included clinical information such as symptoms and signs suggestive of pulmonary tuberculosis (PTB), any previous history of TB treatment, HIV status, absolute CD4 + T cell count, the body mass index as the ratio of weight (kg) and (height)^2^ (m^2^) and socio-demographic characteristics such as age and gender. MDR-TB was treated for at least 20 months and monitored monthly with culture and smear microscopy for AFB while DS TB was monitored with smear microscopy only to determine microbiological treatment response and programmatic outcomes. WGS was performed on baseline MTBC isolates.

### Laboratory procedures

#### Samples collection, culture of MTBC and phenotypic drug-susceptibility testing

Each study participant provided approximately 4 mL of one spot sputum samples for culture. The samples were processed using the modified Petroff’s method [31]. Briefly, 4 mLs of sputum was added to 4 mLs of 4% sodium hydroxide (NaOH). The mixture was vortexed and left to stand at room temperature for 15 min. Thereafter, the volume of the mixture was adjusted to 50 mLs in a falcon tube and concentrated by centrifugation at 3000 g for 15 min. Supernatants were discarded into a container with 25% phenol. Sediments were suspended in Phosphate buffer saline solution (PBS) before being inoculated on LJ culture media as recommended by the Clinical and Laboratory Standard Institute (CLSI) for TB culture [22]. In summary, 200 μL of sputum sediments were inoculated on two slopes of LJ medium containing either pyruvate or glycerol. Culture and identification of MTBC colonies were performed according to locally existing and CLSI standard operating procedures. Phenotypic drug susceptibility testing of the MTBC isolates was performed using a standard proportion method on LJ media [53, 54].

### DNA extraction and whole genome sequencing

Genomic DNA was extracted at Muhimbili University of Health and Allied Sciences (MUHAS) using a modified protocol based on Restriction Fragment Length Polymorphism (RFLP) developed at the National Institute for Public Health and the Environment (RIVM), Bilthoven, Netherlands [55]. The concentration and quality of the DNA were measured using Qubit™ 4 Fluorometer (Invitrogen®). DNA samples were shipped to London School of Hygiene and Tropical Medicine (LSHTM), United Kingdom for whole genome sequencing. The DNA were shipped in screw capped cryo-vials and sealed with parafilm materials. During shipment the DNA were packed in double box and transported in iced packed with insulators materials in a thermo-stable shipping box where the Styrofoam was at least 1.5 in. thick. Library preparation, cluster amplification and sequencing were performed according to the manufacturer’s instructions (QIAGEN). The QIAseq FX DNA Library Kit (QIAGEN) was used for library preparation according to the manufacturer’s protocol. The QIAseq FX DNA Library Kit covers DNA fragmentation for 15 min, adapter ligation and a final purification of the samples using AMPure XP beads (Beckman-Coulter). Library DNA content was analysed using a Qubit 3.0 fluorometer. The sizes of DNA fragments were quantified using the Agilent High Sensitivity DNA Kit (Agilent Technologies) on an Agilent 2100 Bioanalyser (Agilent Genomics) according to the manufacturer’s protocol. Samples were pooled to create pools of libraries at a concentration between 10 and 12pM, and loaded into the MiSeq® using MiSeq® v2 Reagent Kit. WGS was performed at the LSHTM using MiseqTM Sequencing 172 System MiSeqV2–500 cycles (Illumina), producing paired-end sequence reads of 151 bp.

### Bioinformatics and genomic data analysis

#### Quality control and mapping of reads

Initial quality control (QC) reads were characterized using Kraken version v1.1 to exclude contamination and low-quality reads bases of less than 20 Qscore (defined as -10log10 (P), where P is the probability of an error as determined by the sequencing platform). Quality reads were mapped to the H37Rv reference genome (AL123456) using BWA mem [56] with default settings. The median depth and the percentage of the reference covered by a minimum of 10 reads were calculated using SAMtools depth [56] and custom scripts. Samples with sufficient amounts of MTBC data were used for downstream analysis.

#### Variant calling and in-silico resistance and lineage typing

Variants in drug resistance gene candidates were called using LoFreq version v2.1.2 [57]. These were annotated and compared to the TBProfiler database [58] (http://tbdr.lshtm.ac.uk/) as it does not perform its own variant calling but annotates existing calls as drug resistance mutations using a database of mutations. Additionally, large indels were identified by looking at depth in candidate genes. The coverage across lineage specific markers was inspected using htsbox (https://github.com/lh3/htsbox) and custom scripts to assign specific lineages to the isolates.

#### Phylogeny reconstruction

Variants throughout the whole genome were called using SAMtools/BCFtools version v1.9 [59]. The resulting variant call format (VCF) files were collated together filtering was performed. In short, sites in samples were marked as missing if less than 5 reads aligned to a position and monomorphic sites or those with greater than 10% missing data were removed. Using all SNPs, a maximum likelihood phylogeny was constructed using ExaML version v3.0.21, a state of the art tool for phylogenomic analyses [60]. The phylogenetic reconstruction was used in conjunction with lineage predictions output by TBProfiler. These predictions were based on the universally adopted lineage nomenclature and SNP barcode reported by Coll et al. [61]. This was visualized with drug resistance phenotypes and lineages using iTOL [62]. The lineages were assigned based on the SNP barcode available at doi:10.1038/ncomms5812 and has been adopted as the standard for Mtb WGS data.

### Analysis of clinical data

Descriptive statistics was performed for demographic characteristics, frequency and distribution of mutation patterns in MDR-TB and other resistance patterns in MTBC. A Pearson chi-square test was used to determine the association between age, gender, HIV status and CD4 counts if applicable, Body mass index (BMI), history of previous TB treatment and genotype drug resistance as the main outcome using STATA version 14 (StataCorp LP, College Station, TX, USA). A *p*-value of < 0.05 considered statistically significant. Genotype drug resistance was defined as any mutations that are known to confer decreased anti tuberculosis drug susceptibility. Skewed data, Student’s -test and Wilcoxon rank-sum tests were used for normally distributed data. The body weight based on BMI values for both adults was categorized into two groups namely; thinness (≤ 18.5) and normal> 18.5 as recommended by the World Health Organization (WHO). Kappa statistics (K) were used to determine agreement between drug susceptibility testing (DST) and genetic drug resistance using four levels of agreement for kappa: < 0.40 (poor), 0.40–0.59 (fair), 0.60–0.80 (good), and > 0.80 (excellent) [63].

## Data Availability

Raw sequence data are available from the European Nucleotide Archive (ENA) under the project accession number PRJEB29435 (https://www.ebi.ac.uk/ena/data/view/PRJEB29435).

## Abbreviations

AIDS: Acquired immunodeficiency syndrome
BMI: Body mass index
DR-TB: Drug resistant tuberculosis
DS-TB: drug susceptible tuberculosis
HIV: Human immunodeficiency virus
KIDH: Kibong’oto infectious diseases hospital
LJ: Lowenstein jensen
MDR-TB: Multidrug resistant Tuberculosis
MTB: *Mycobacterium tuberculosis*
PTB: Pulmonary tuberculosis
SNP: Single nucleotide polymorphism
TB: Tuberculosis
WHO: World health organization
XDR-TB: Extensive DR-TB
